# Investigating the effects of percussion massage therapy on pain, functionality, muscle diameter, and proprioception in ındividuals with ACL reconstruction: a randomized controlled trial

**DOI:** 10.1371/journal.pone.0319731

**Published:** 2025-03-26

**Authors:** Beyza Nur Erayata, Burak Menek

**Affiliations:** 1 Department of Physiotherapy and Rehabilitation, Institute of Health Sciences, Istanbul Medipol University, Istanbul, Turkey; 2 Department of Physiotherapy and Rehabilitation, Faculty of Health Sciences, Istanbul Medipol University, Istanbul, Turkey; Erzurum Technical University: Erzurum Teknik Universitesi, TÜRKIYE

## Abstract

**Background:**

Percussion massage therapy (PMT) integrates traditional massage with vibration therapy. This study examined the effects of adding PMT to a structured exercise program for individuals who underwent surgery after an anterior cruciate ligament (ACL) injury.

**Methods:**

A total of 24 individuals aged 18–40 were included in the study. Participants were divided into the PMT and structured exercise groups (SEG). The SEG group received a progressive neuromuscular exercise program, and the PMT group received the same structured exercise program and PMT. Range of motion (ROM) was assessed using the Goniometer Pro smartphone application, joint position sense (JPS) was measured goniometer, pain intensity was evaluated with the Visual Analog Scale (VAS), functionality was assessed using the Western Ontario and McMaster Universities Osteoarthritis Index (WOMAC), balance was measured with the Berg Balance Scale, quality of life was evaluated using the Short Form-36 (SF-36), and muscle diameter was measured via ultrasonography.

**Results:**

Significant improvements were observed in all parameters in both groups post-treatment (p < 0.05). The PMT group showed superior results compared to the SEG group in ROM, JPS (60°), pain, functionality, balance, and quality of life (particularly in the general health perception sub-parameter of SF-36) (p < 0.05).

**Conclusions:**

The findings of this study suggest that incorporating PMT, a novel approach in the literature, into the rehabilitation program following ACL reconstruction could be effective. PMT could be an alternative treatment method that can be used in conjunction with exercise programs in ACL rehabilitation.

**Trial Registiration:**

NCT06185231.

## Introduction

An anterior cruciate ligament (ACL) rupture is a prevalent injury in sports that involves cutting, jumping, or pivoting. The majority of individuals with an ACL injury require ACL reconstruction surgery to restore the knee stability needed for returning to sports participation [1]. Following ACL reconstruction, several months of supervised rehabilitation are necessary [2]. Following an ACL injury, treatment options include conservative management or surgical intervention. The primary goals of both treatment approaches are to stabilize the knee, restore normal joint movement, enhance muscular strength around the hip and knee, and improve proprioception [3,4]. Pain management is a critical component of ACL rehabilitation, as pain can limit range of motion, hinder functional recovery, and negatively impact the overall quality of life of individuals undergoing rehabilitation. Effective pain control is essential for achieving optimal outcomes in rehabilitation programs [5,6]. Vibration therapy has gained popularity in rehabilitation over the past decade. This therapy can be administered using whole-body vibration (WBV) platforms or locally with percussion massage devices [7]. Percussion massage therapy (PMT) has become a frequently used myofascial intervention in recent years, aiming to reduce pain in both deep and superficial tissues, increase blood circulation, improve scar tissue healing, decrease lactate levels and muscle spasms, enhance lymphatic flow, inhibit the Golgi Tendon Reflex, and increase range of motion (ROM) [8].

Percussion massage devices have recently gained popularity for therapeutic use and among athletes. These devices provide local vibration therapy (LVT) by combining elements of traditional massage and vibration therapy. It is believed that they enhance flexibility and performance, thereby accelerating recovery. Despite their growing popularity, the definitive effects of these devices have yet to be conclusively proven [9]. Percussion massage devices, typically powered by electricity or batteries, are frequently employed in PMT to deliver rapid compressive forces to the myofascia using a variety of attachment shapes. Several manufacturers, including Theragun and Hypervolt, produce these devices for both self-massage and professional use by physiotherapists. These devices can function at varying frequencies, with some reaching up to 53 Hz [10]. To effectively target specific local points depending on the tissue type, different attachment heads can be utilized with these devices (Hypervolt, Hyperice, California, US) [11]. A review of the literature on PMT reveals that studies have predominantly focused on healthy individuals and athletes. These studies have primarily examined the effects of PMT on flexibility, muscle strength, and ROM [12]. A review of the literature reveals that studies on PMT and ACL reconstruction are limited [12,13]. A study examining the effectiveness of WBV in ACL rehabilitation reported that WBV was more effective than conventional exercise methods in improving balance parameters. However, both methods provided similar effects on muscle strength and functionality parameters [14]. In a study conducted by Pistone et al., it was reported that WBV was more effective in increasing knee flexor muscle strength compared to the control group after ACL reconstruction, but it did not show any superiority in balance parameters [15].

Our study hypothesized that PMT, when added to a structured exercise program, could be more effective in ACL rehabilitation on parameters such as pain, range of motion, joint position sense, balance, quality of life, and functionality. This study aimed to investigate the effects of PMT on pain, functionality, quality of life, muscle diameter, balance, and joint position sense in individuals who have undergone ACL surgery and compare the superiority of PMT over a structured exercise program.

## Methods

### Study design and participants

This study is a single-blind, randomized trial in which participants were assigned to one of two groups in a 1:1 ratio. The trial received approval from the Non-interventional Ethics Committee at Istanbul Medipol University (Approval Number: E-10840098-772.02-6782). All participants provided written informed consent, and the study adhered to the principles of the Declaration of Helsinki. The study protocol was registered on ClinicalTrials.gov (NCT06185231). Our study protocol and ethical approval were thoroughly prepared and submitted to the ethics committee. However, due to operational requirements, the patient recruitment process had to commence earlier than the planned registration period. Despite this, our study was conducted in full compliance with international ethical guidelines and clinical research standards. The authors confirm that all ongoing and related trials for this intervention are registered. This study commenced on September 15, 2023, and was completed on June 30, 2024.

**Fig 1:**
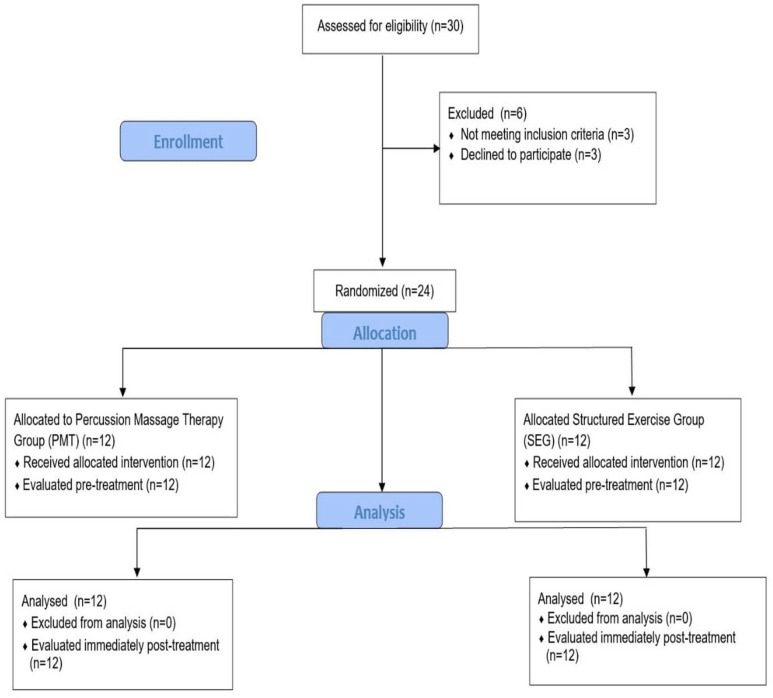
Design and flow chart of the study.

The study included thirty individuals aged 18-40 who had undergone ACL reconstruction at Esenler Medipol University Hospital. The study included individuals aged twenty to forty years who had undergone ACL reconstruction using a hamstring tendon autograft, had a unilateral full-thickness ACL tear, and presented to the physical therapy clinic within the first eight weeks following surgery. Exclusion criteria were a history of secondary ACL reconstruction, the presence of musculoskeletal, neurological, or vestibular disorders affecting the lower extremities, chronic or systemic conditions that could lead to peripheral neuropathy or proprioceptive deficits (such as diabetes or chronic renal failure), as well as cognitive, mental, or psychological issues. Additionally, individuals with balance impairments, those taking medications that might negatively influence balance, or those diagnosed with other knee-related conditions were also excluded from the study. Three participants declined to participate in the study, and three others were excluded for not meeting the inclusion criteria. The remaining eligible participants were randomized into two groups: the structured exercise group (SEG) (n = 12) and the PMT group (n = 12) (Fig 1). Randomization was conducted by placing 24 pieces of paper into a closed box, with half labeled with odd numbers and the other half with even numbers. Each participant enrolled in the study randomly selected a piece of paper from the box. Participants who drew an odd number were assigned to the SEG group, while those who drew an even number were allocated to the PMT group. To minimize bias, participants were blinded to their group assignments and were not informed about the interventions applied to the other group

#### Structured exercise group.

**Fig 2:**
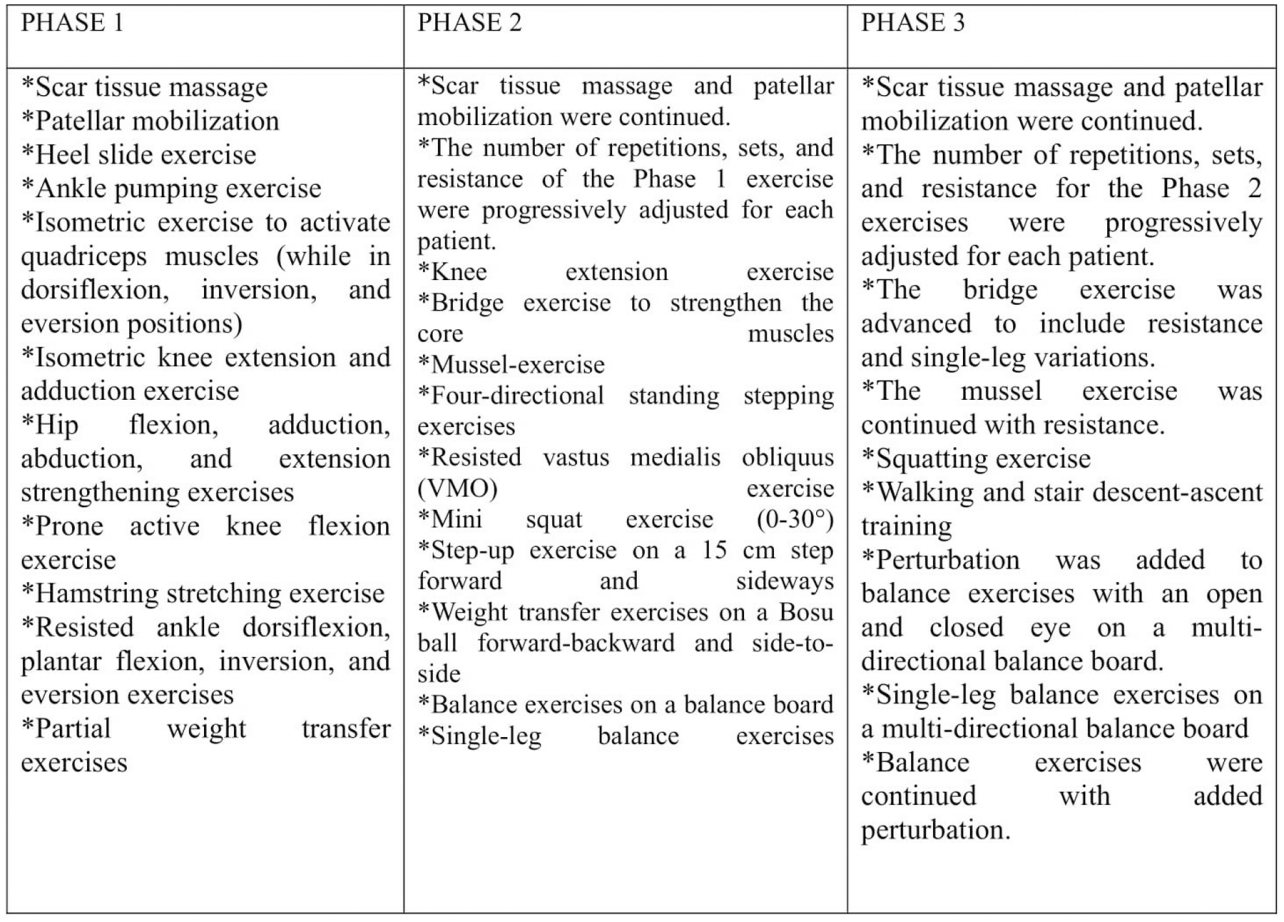
Structured Exercise Program.

Participants underwent thirty treatment sessions over six weeks, with sessions held five days per week. Assessments were conducted before and after the treatment. Participants in this group followed a progressive exercise program outlined in three phases (Fig 2). The structured exercise program was designed to progress through three phases, each targeting different aspects of recovery, including ROM, strength, balance, and proprioception. The progression was individualized and tailored to each participant’s tolerance and recovery status, as monitored by the supervising physiotherapist. In Phase 1, the focus was on restoring basic ROM, reducing swelling, and activating the quadriceps through isometric exercises. The intensity of exercises was kept low to ensure safe initiation of movement. In Phase 2, the intensity, repetitions, and resistance of exercises were gradually increased. This phase introduced more dynamic exercises, including weight-bearing and stepping activities, to enhance strength and balance. In Phase 3, exercises were further advanced to include additional resistance, single-leg variations, and perturbation challenges to improve functional stability and proprioception. Progression within each phase was based on the participant’s ability to perform exercises without pain or discomfort, ensuring a safe and effective recovery process. Adjustments were made by increasing the number of repetitions, resistance levels, or complexity of exercises, as appropriate for each individual.

#### Percussion massage therapy group.

In addition to the structured exercise program, participants in the PMT group also received PMT. The therapy was administered by the same researcher using a Hypervolt device (Hyperice, CA, USA). Treatment was applied to both the quadriceps and hamstring muscle groups prior to exercise. Using the softball head attachment on the percussion device, the massage was delivered at a frequency of 40 Hz for a total duration of 5 minutes per muscle group. For the initial 2.5 minutes, the therapy was applied to the medial side of the muscle, followed by 2.5 minutes on the lateral side. The application began with the medial side of the muscle, following the origin-to-insertion line over 5 seconds. This procedure was then repeated on the lateral side of the muscle. Thus, the PMT for each muscle began on the medial side and concluded on the lateral side [12,16]. After the study, participants who did not receive PMT were asked if they would like to undergo the treatment. PMT was administered after the study for those who expressed interest.

### Outcome measures

Outcome assessments for all participants were conducted both prior to the treatment and after six weeks of treatment.

#### Assessment of range of motion.

The range of motion was assessed using the Goniometer Pro smartphone application. Patients were positioned prone, and 0 0-degree full knee extension was established. The smartphone was placed on the midline of the lateral tibia, and patients were asked to actively flex their knee. The angular data was recorded at the endpoint of the active flexion movement. Measurements were taken three times in total, and the average value was calculated. The Goniometer Pro smartphone application has been demonstrated to have high reliability and concurrent validity for measuring knee angles, as shown in previous studies [17,18].

#### Joint position sense.

Joint position sense was assessed using the angle reproduction test with a goniometer. The assessment was conducted at two target angles, 60 degrees, and 30 degrees, with the starting angle considered to be 90 degrees. Initially, with the knee joint at 90 degrees flexion, the participant’s knee was passively moved to the first target angle of 60 degrees flexion and held in that position for 5 seconds. The participant was asked to remember this angle and, during the test, to hold their knee at the same angle when they believed they had reached it. This procedure was repeated three times with the eyes open and three times with the eyes closed before the test. During the test, the participant’s knee was returned to the starting position of 90 degrees, and they were asked to actively flex their knee to the 60-degree position. The flexion angle at which the participant held their knee was recorded. The same procedure was repeated for the other target angle [19].

#### Pain.

Our study assessed the participants’ pain levels under two subcategories: at rest and during activity. The Visual Analog Scale (VAS) was used to evaluate pain. The Visual Analog Scale is used to measure pain intensity and is self-reported by the participant. Participants were asked to mark a point on a line ranging from 0 to 10 that represented their pain intensity by drawing a perpendicular line on the VAS scale. On this scale, a value of 0 indicates no pain, while a value of 10 represents unbearable pain. A higher score indicates greater pain intensity [20].

#### Balance.

In this study, balance parameters were assessed using the Berg Balance Scale. The scale is composed of 14 different tasks that evaluate various functions, including rising from a seated position, sitting unsupported, standing unsupported, sitting down from a standing position, standing with eyes closed, transferring between positions, standing with feet together, picking an object up from the floor, reaching forward while standing, looking over each shoulder, turning 360 degrees, standing on one leg, standing with one foot in front of the other, and maintaining balance on one leg [21].

#### Functionality.

The functional status of the knee joint was evaluated using the Western Ontario and McMaster Universities Osteoarthritis Index (WOMAC), a widely accepted and reliable tool for assessing physical function in individuals who have undergone knee surgery. The scale includes 24 questions in total: five questions addressing pain (0–20 points), two questions focused on stiffness (0–8 points), and 17 questions measuring difficulty with physical function (0–68 points). Higher WOMAC scores reflect a greater level of impairment [22].

#### Quality of life.

The Short Form-36 (SF-36) scale was used to evaluate the quality of life of participants. This 36-item questionnaire assesses eight domains related to both physical and mental health components. Each item is scored and combined following a standardized scoring method. Scores for each domain range from 0 to 100, with higher scores representing better health status [23].

#### Muscle diameter.

Muscle diameter measurements were obtained using a G.E. Logiq P5 Ultrasound Device (GE Medical Systems, Milwaukee, WI, USA) (5–13 MHz). All measurements were conducted by a single researcher. Participants were positioned supine to prevent hip external rotation. To balance fluid shifts, participants were assessed after lying supine with both legs fully extended for 10 minutes. A water-based gel was applied between the ultrasound probe and the skin to enhance acoustic contact and prevent inaccurate measurements. The quadriceps muscles were imaged in the following order: rectus femoris (RF), vastus lateralis (VL), and vastus medialis (VM). An anthropometric line was marked extending from the superior edge of the patella to the anterior superior iliac spine (ASIS) to determine the measurement points for the quadriceps muscles. The RF was measured at 50% of this line. For VL, the thigh circumference was taken at 50% of the line, and the VL was measured laterally at 10% of the circumference distance from the line. The VM was measured at 20% of the line, and the thigh circumference was measured at this point. The VM was measured medially at 12.5% of the circumference distance from the line [24].

**Fig 3:**
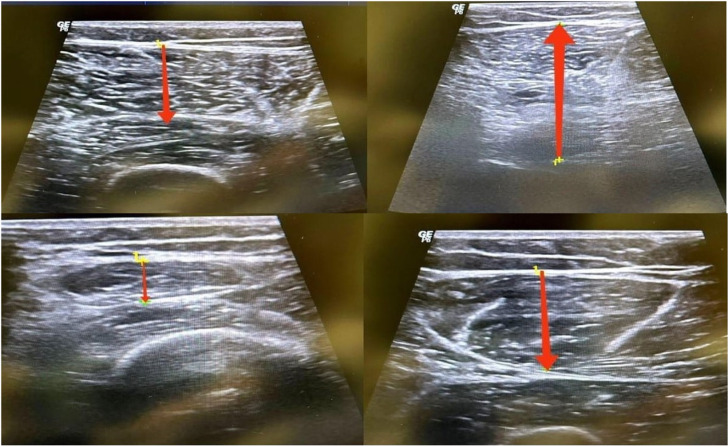
Measurement of Muscle Diameter.

The measurement of the biceps femoris long head (BFlh) was used to represent the hamstring muscles. The measurement was recorded with participants lying prone, with the hip and knee in full extension. The BFlh muscle-tendon complex was identified on the skin through palpation and isometric contractions. The length between the origin and insertion of the BFlh was then measured. The ultrasound scan involved moving the probe continuously and steadily along the marked line from the insertion of the BFlh over 3-5 seconds. During the scan, the probe was adjusted to remain perpendicular to the skin and aligned with the fascicle direction of the BFlh. Care was taken to minimize the pressure exerted by the probe on the skin to avoid affecting the accuracy of the measurements [25] (Fig 3).

#### Statistical analysis.

Data were analyzed using IBM SPSS Statistics Standard Concurrent User Version 26 (IBM Corp., Armonk, New York, USA). Descriptive statistics were expressed as number of units (n), percentage (%), mean (X), standard deviation (SD), median (M), and minimum (min) and maximum (max) values. In the decision-making process, if the absolute skewness value was less than ±2.0 and the kurtosis value was under 7.0, the data were considered to follow a normal distribution. The skewness and kurtosis values of the study variables indicated normality. Consequently, parametric tests were applied as both the descriptive numerical characteristics and variables demonstrated normal distribution. Chi-square tests (Pearson chi-square/Fisher’s exact test) were employed to analyze the distribution of categorical variables across the groups. The Independent Samples t-test was utilized to compare the numerically measured demographic variables and the differences between pre- and post-treatment measurements between the groups. For within-group comparisons before and after treatment, the Paired Samples t-test was applied. A p-value of <0.05 was regarded as statistically significant, and Cohen’s d was used to calculate the effect size.

## Results

In this study, a total of 24 participants were included, with 12 in the PMT group and 12 in the SEG group. The demographic characteristics of the participants in both the PMT and SEG groups were comparable (p > 0.05). The distribution of demographic characteristics according to the groups is presented in Table 1. The pretreatment evaluation parameters of the groups are presented in Table 2. Pretreatment VAS-rest, VAS-activity, and WOMAC scores were significantly higher in the PMT group compared to the SEG group, whereas Rectus Femoris, Vastus Lateralis, and Vastus Medialis measurements were lower in the PMT group than in the SEG group (p < 0.05). Other evaluation parameters were similar between the two groups (p > 0.05). When the pre-treatment and post-treatment values of the PMT and SEG groups were examined, a significant improvement was observed in all parameters (p < 0.05) (Table 3). The differences within groups before and after treatment and the comparison of these differences between groups are shown in Table 4. In the comparison of differences between groups, the PMT group showed significant results compared to the structured exercise group in terms of flexion and extension range of motion, 60° Joint Position Sense, VAS-resting, VAS-activity, WOMAC, Berg Balance Scale, and the General Health sub-parameter of SF-36 (p < 0.05).

**Table 1:** Distribution of the demographic and physical information.

Variables	PMT (mean±SD)	SEG (mean±SD)	p-value
**Age (years)** (Min-Max)	27.75 ± 7.07 (18–39)	28.42 ± 7.73 (19–40)	0.828
**Height (cm)** (Min-Max)	176.17 ± 5.34 (167–182)	179.58 ± 5.81 (170–194)	0.148
**Weight(kg)** (Min-Max)	85.67 ± 14.38 (67–115)	83.08 ± 14.18 (59–115)	0.662
**Body Mass Index** (Min-Max)	27.63 ± 4.87 (22.6–37.2)	25.84 ± 5.10 (18.8–39.8)	0.139
**Time to start rehabilitation (Weeks)** (Min-Max)	6 ± 1.35 6 (4–8)	6 ± 1.81 5 (4–8)	0.999

PMT: Percussion massage therapy group; SEG: Structured exercise group; SD: Standard deviation; kg: kilogram; cm: centimeter.

**Table 2:** Comparison of pretreatment measurements between groups.

Variables	PMT Pre treatment (mean ± SD)	SEG Pre treatment (mean ± SD)	p-value
ROM/Flexion	85.25 ± 16.01	94.42 ± 15.30	0.166
ROM/Extansion	10.00 ± 4.26	7.08 ± 5.82	0.175
JPS-60°	65.83 ± 3.24	63.58 ± 3.00	0.091
JPS-30°	36.00 ± 2.70	37.17 ± 3.46	0.367
VAS-Resting	6.17 ± 1.40	4.75 ± 1.22	0.015
VAS-Activity	8.25 ± 0.87	7.42 ± 0.90	0.031
WOMAC	82.11 ± 14.75	54.94 ± 22.55	0.002
Berg Balance Test	30.50 ± 10.75	38.58 ± 11.53	0.090
SF-36/ Physical Function	30.00 ± 21.64	35.42 ± 16.71	0.500
SF-36/ Role Physical	16.67 ± 34.27	18.75 ± 38.62	0.890
SF-36/ Bodily Pain	39.50 ± 23.29	33.67 ± 20.52	0.522
SF-36/ General Health	53.75 ± 13.34	62.50 ± 17.39	0.180
SF-36/ Vitality	37.5 ± 15.45	44.17 ± 22.24	0.403
SF-36/ Social Function	22.08 ± 20.10	35.75 ± 25.92	0.163
SF-36/ Role Emotional	33.25 ± 37.63	27.67 ± 37.14	0.718
SF-36/ Mental Health	52.00 ± 11.44	54.00 ± 20.78	0.773
Muscle Diameter-RF	2.14 ± 0.25	2.34 ± 0.20	0.040
Muscle Diameter-VL	1.75 ± 0.29	2.07 ± 0.28	0.010
Muscle Diameter-VM	1.53 ± 0.33	1.90 ± 0.28	0.008
Muscle Diameter-BF	3.50 ± 0.38	3.51 ± 0.39	0.975

PMT: Percussion massage therapy group; SEG: Structured exercise group; ROM: Range of motion; JPS: Joint position sense; VAS: Visual analog scale; WOMAC: Western Ontario and McMaster Universities Osteoarthritis Index; SF-36: Short form-36; RF: Rectus Femoris; VL: Vastus Lateralis; VM: Vastus Medialis; BF: Biceps Femoris; SD: Standart deviation.

**Table 3:** Comparison of changes in outcome measures within and between the groups.

Variables	PMT (mean±SD)		p-value	Cohen d	SEG (mean±SD)		p-value	Cohen d
	Pre	Post			Pre	Post		
ROM/Flexion	85.25 ± 16.01	128.33 ± 3.26	<0.001	−3.100	94.42 ± 15.30	124.58 ± 6.56	<0.001	−1.866
ROM/Extansion	10.00 ± 4.26	0.42 ± 1.44	<0.001	−2.417	7.08 ± 5.82	0.83 ± 1.95	<0.001	−1.184
JPS-60°	65.83 ± 3.24	60.00 ± 0.85	<0.001	1.686	63.58 ± 3.00	60.75 ± 1.42	<0.001	1.142
JPS-30°	36.00 ± 2.70	30.33 ± 0.78	<0.001	2.119	37.17 ± 3.46	32.33 ± 1.87	<0.001	1.253
VAS-Resting	6.17 ± 1.40	0.42 ± 0.67	<0.001	4.043	4.75 ± 1.22	0.58 ± 0.67	<0.001	4.445
VAS-Activity	8.25 ± 0.87	1.33 ± 1.07	<0.001	6.943	7.42 ± 0.90	1.92 ± 0.90	<0.001	5.059
WOMAC	82.11 ± 14.75	10.41 ± 7.17	<0.001	4.460	54.94 ± 22.55	8.85 ± 5.30	<0.001	2.287
Berg Balance Test	30.50 ± 10.75	55.17 ± 1.8	<0.001	−2.489	38.58 ± 11.53	54.08 ± 4.19	<0.001	−1.451
SF-36/ Physical Function	30.00 ± 21.64	88.33 ± 11.35	<0.001	−2.605	35.42 ± 16.71	85.83 ± 8.21	<0.001	−2.558
SF-36/ Role Physical	16.67 ± 34.27	75.00 ± 26.11	<0.001	−1.558	18.75 ± 38.62	77.08 ± 32.78	<0.001	−1.397
SF-36/ Bodily Pain	39.50 ± 23.29	77.08 ± 6.05	<0.001	−1.461	33.67 ± 20.52	70.00 ± 18.27	<0.001	−1.279
SF-36/ General Health	53.75 ± 13.34	81.67 ± 5.77	<0.001	−1.769	62.50 ± 17.39	78.75 ± 14.64	<0.001	−1.519
SF-36/ Vitality	37.5 ± 15.45	66.25 ± 14.00	<0.001	−2.518	44.17 ± 22.24	73.75 ± 21.01	<0.001	−1.106
SF-36/ Social Function	22.08 ± 20.10	65.75 ± 10.79	<0.001	−1.894	35.75 ± 25.92	74.25 ± 24.64	<0.001	−1.846
SF-36/ Role Emotional	33.25 ± 37.63	86.17 ± 22.28	<0.001	−1.709	27.67 ± 37.14	80.58 ± 33.23	<0.001	−1.094
SF-36/ Mental Health	52.00 ± 11.44	73.67 ± 8.08	<0.001	−2.034	54.00 ± 20.78	79.33 ± 15.71	<0.001	−1.658
Muscle Diameter-RF	2.14 ± 0.25	2.27 ± 0.26	<0.001	−1.644	2.34 ± 0.20	2.45 ± 0.25	<0.001	−1.695
Muscle Diameter-VL	1.75 ± 0.29	1.88 ± 0.31	<0.001	−1.947	2.07 ± 0.28	2.16 ± 0.28	<0.001	−2.093
Muscle Diameter-VM	1.53 ± 0.33	1.64 ± 0.35	<0.001	−1.911	1.90 ± 0.28	1.98 ± 0.27	<0.001	−1.262
Muscle Diameter-BF	3.50 ± 0.38	3.65 ± 0.37	<0.001	−2.599	3.51 ± 0.39	3.65 ± 0.42	<0.001	−1.572

PMT: Percussion massage therapy group; SEG: Structured exercise group; ROM: Range of motion; JPS: Joint position sense; VAS: Visual analog scale; WOMAC: Western Ontario and McMaster Universities Osteoarthritis Index; SF-36: Short form-36; RF: Rectus Femoris; VL: Vastus Lateralis; VM: Vastus Medialis; BF: Biceps Femoris; SD: Standart deviation.

**Table 4:** Differences within groups before and after treatment and comparison of differences between groups.

Variables	PMT (mean±SD)	SEG (mean±SD	p-value	Cohen d
ROM/Flexion	43.08 ± 13.9	30.17 ± 16.16	0.048	0.857
ROM/Extansion	9.58 ± 3.96	6.25 ± 5.28	0.044	0.714
JPS-60°	5.83 ± 3.46	2.83 ± 2.48	0.023	0.997
JPS-30°	5.67 ± 2.67	4.83 ± 3.86	0.545	0.253
VAS-Resting	5.75 ± 1.42	4.17 ± 0.94	0.004	1.312
VAS-Activity	6.92 ± 1.00	5.50 ± 1.09	0.003	1.358
WOMAC	71.7 ± 16.07	46.09 ± 20.15	0.002	1.405
Berg Balance Test	24.67 ± 9.91	15.50 ± 10.68	0.040	0.890
SF-36/ Physical Function	58.33 ± 22.39	50.42 ± 19.71	0.368	0.375
SF-36/ Role Physical	58.33 ± 37.44	58.33 ± 41.74	0.999	0.000
SF-36/ Bodily Pain	37.58 ± 21.99	36.33 ± 33.20	0.914	0.044
SF-36/ General Health	27.92 ± 13.73	16.25 ± 9.8	0.026	0.978
SF-36/ Vitality	28.75 ± 16.25	29.58 ± 19.48	0.910	−0.046
SF-36/ Social Function	43.67 ± 23.05	38.5 ± 20.85	0.571	0.235
SF-36/ Role Emotional	52.92 ± 36.21	52.92 ± 41.39	0.999	0.000
SF-36/ Mental Health	21.67 ± 8.61	25.33 ± 22.90	0.609	−0.212
Muscle Diameter-RF	0.14 ± 0.08	0.11 ± 0.07	0.437	0.399
Muscle Diameter-VL	0.13 ± 0.07	0.09 ± 0.04	0.066	0.702
Muscle Diameter-VM	0.1 ± 0.05	0.08 ± 0.06	0.328	0.362
Muscle Diameter-BF	0.15 ± 0.06	0.14 ± 0.09	0.765	0.131

PMT: Percussion massage therapy group; SEG: Structured exercise group; ROM: Range of motion; JPS: Joint position sense; VAS: Visual analog scale; WOMAC: Western Ontario and McMaster Universities Osteoarthritis Index; SF-36: Short form-36; RF: Rectus Femoris; VL: Vastus Lateralis; VM: Vastus Medialis; BF: Biceps Femoris; SD: Standart deviation.

## Discussion

Upon examining the study’s results, within-group comparisons for the PMT and SEG groups revealed improvements in all parameters post-treatment. When comparing groups, the PMT group demonstrated more effective improvements than the SEG group in terms of range of motion, joint position sense, pain, functionality, balance, and the general health perception sub-parameter of quality of life.

ACL injuries are among the most common and debilitating knee injuries, particularly in athletic activities. These injuries result in joint instability, muscle weakness, and pain, necessitating a rehabilitation program aimed at restoring function and minimizing disabilities. Effective rehabilitation focuses on reducing pain and edema, improving range of motion, strength, and endurance, and enhancing proprioception and dynamic stability, with careful consideration of knee biomechanics to optimize outcomes [3,26]. Upon reviewing the literature, it is evident that rehabilitation protocols following ACL injuries typically emphasize neuromuscular training during the first three months post-surgery. Neuromuscular training includes exercises designed to develop rapid and optimal muscle contraction patterns. These programs aim to enhance dynamic joint stability and facilitate relearning movement patterns and skills necessary for daily activities and sports [27]. In the study by Harput et al. investigating the effectiveness of neuromuscular training after ACL reconstruction, it was reported that neuromuscular training led to an increase in hamstring and quadriceps muscle strength in both the affected and unaffected extremities at the 4th, 8th, and 12th weeks of muscle strength assessments [28]. Risberg et al. reported that a 6-month neuromuscular training program following ACL reconstruction was more effective in reducing knee pain than a traditional strength training program. The results of this study suggest that exercises included in neuromuscular training programs should be an integral part of post-ACL reconstruction rehabilitation to help reduce pain severity [29]. In our study, we implemented a progressive, structured neuromuscular rehabilitation program tailored to the functional status of the individuals. Consistent with the literature, our study supports the view that prioritizing neuromuscular training in the planning of rehabilitation programs following ACL injury can lead to better outcomes in patients’ recovery processes.

Vibration therapy, including the use of percussion massage guns, has been explored for its effects on physical performance and pain. These devices deliver vibrations at varying frequencies, offering a practical approach to rehabilitation. Research has shown that vibration therapy can lead to acute benefits, such as pain reduction, increased strength, and enhanced flexibility, following one or multiple treatments. However, despite the extensive body of literature on vibration therapy, there is limited research specifically focusing on the physiological adaptations associated with PMT using massage guns [30–32]. Upon reviewing the literature, it is evident that the majority of studies on PMT have been conducted on healthy or athletic individuals, with findings indicating that PMT effectively increases flexibility in this population [9,12,33,34]. Sams et al. demonstrated that PMT, applied using mechanical percussion devices, is effective in enhancing flexibility and reducing musculoskeletal pain [12]. Furthermore, it has been reported that PMT, when combined with conventional physiotherapy, improves pain, joint position sense, and functionality, leading to its recommendation as an adjunct treatment option in rehabilitation programs [35]. In contrast to the existing literature, our study focused on individuals with ACL injuries rather than healthy or athletic participants. By addressing this gap, our research adds to the limited body of evidence on the use of PMT in pathological conditions. The findings of our study indicate that incorporating PMT into structured exercise programs enhances ROM, functionality, and joint position sense. Additionally, consistent with previous research, our results suggest that PMT contributes to improved muscle flexibility, providing significant benefits in the context of ACL rehabilitation. We believe that the application of PMT reduces muscle tension, thereby increasing blood circulation and promoting fascial release, which in turn leads to an improvement in ROM. In the study conducted by Berschin et al., it was reported that eight weeks of vibration therapy improved postural stability and functionality in individuals who underwent ACL reconstruction [14]. Pistone et al. investigated the short-term effects of WBV training on maximum strength and balance following ACL reconstruction. The study found that vibration training was more effective in increasing muscle strength, but no significant differences were observed between the groups in terms of balance parameters [15]. In our study, the improvement in balance measurements was observed to be superior in the PMT group compared to the SEG group. We believe that this improvement may be attributed to the longer duration of the intervention. Additionally, we consider that the vibration stimulus applied through PMT may have positively influenced the proprioceptive system, thereby contributing to enhanced balance reactions. This finding aligns with the literature highlighting the neuromuscular control-supporting effects of vibration therapy. Tankisheva et al. investigated the effects of local vibration therapy on bone mineral density, muscle mass, and physical performance in women. Vibration therapy was applied for six months, five days a week, within a frequency range of 30-45 Hz. The results showed that vibration therapy increased isometric muscle strength but had no significant effect on muscle mass or physical performance [36]. In our study, similar results were obtained with PMT applied at a frequency of 40 Hz. Muscle thickness was measured using ultrasonography, and the increase in muscle thickness was associated with the increased muscle strength achieved through exercise. The results of our study showed that the increase in quadriceps and hamstring muscle thickness exhibited a similar effect in both the PMT and SEG groups. Consistent with the findings of Tankisheva et al., our study also demonstrated that the effectiveness of PMT in increasing muscle thickness was similar to that of the structured exercise program. Percussion massage devices have emerged as a novel treatment method that is increasingly used in sports and clinical settings. The lack of evidence-based reviews and clinical guidelines to inform practice is particularly notable. Specifically, there is insufficient information regarding how these devices should be applied by healthcare professionals, including which attachment to use, how to apply the treatment, the effects at various frequency ranges, and the appropriate duration of application [10]. To date, the majority of published studies have focused on the effects of PMT on joint range of motion and muscle stiffness. However, studies investigating the effects of PMT on musculoskeletal problems are quite limited in the literature [12].

We consider one of the strengths of our research to be the investigation of the effectiveness of PMT in individuals with ACL injuries, along with the objective measurement of muscle thickness. However, the study has certain limitations. The primary limitation is the small sample size. Additionally, the long-term outcomes of the interventions were not assessed in this study. Another limitation is the significant differences observed in certain pretreatment parameters, including VAS-rest, VAS-activity, WOMAC scores, and muscle diameter measurements, between the PMT and SEG groups. Although these differences were statistically controlled in the analysis, they may have influenced the interpretation of the treatment effects. Future studies should aim to include larger participant groups, extend the follow-up duration, and examine different frequencies and durations of PMT to further confirm these findings, address baseline imbalances, and gain a deeper understanding of the long-term effects and optimal parameters of PMT.

## Conclusions

This study has demonstrated that both PMT and neuromuscular-based exercise programs are effective in improving pain, range of motion, joint position sense, balance, and quality of life in individuals with ACL injuries. The addition of PMT to the exercise program resulted in superior outcomes in terms of ROM, joint position sense, pain, functionality, and balance parameters compared to the group that received only exercise. Our findings suggest that PMT could be an alternative treatment method that can be used in conjunction with exercise programs in ACL rehabilitation and may provide significant benefits during the rehabilitation process.
